# Time-to-event ensemble machine learning approach for predicting long-term survival of abdominal aortic aneurysm patients undergoing endovascular aneurysm repair

**DOI:** 10.1371/journal.pone.0349122

**Published:** 2026-06-12

**Authors:** Hong-Jae Choi, Changhee Lee, Joon Seo Lim, You Jung Ok, Jae-Sung Choi, Jae Hwa Jeong, Yong Won Seong, Hyeon Jong Moon, Se Jin Oh

**Affiliations:** 1 Department of Thoracic and Cardiovascular Surgery, SMG-SNU Boramae Medical Center, Seoul National University College of Medicine, Seoul, Republic of Korea; 2 Department of Artificial Intelligence, Korea University, Seoul, Republic of Korea; 3 Clinical Research Center, Asan Medical Center, University of Ulsan College of Medicine, Seoul, Republic of Korea; 4 Healthcare AI Research Institute, Seoul National University Hospital, Seoul, Republic of Korea; Karmanos Cancer Institute, Wayne State University School of Medicine, UNITED STATES OF AMERICA

## Abstract

**Background:**

Endovascular aneurysm repair (EVAR) for abdominal aortic aneurysm (AAA) is associated with risks such as endoleaks and late aneurysm rupture, highlighting the importance of long-term survival prediction. Despite recent advancements in machine learning (ML), predictive models utilizing time-to-event analysis remain limited for AAA patients undergoing EVAR. We aimed to develop a stacking ensemble ML model to predict long-term outcomes in EVAR-treated AAA patients.

**Methods:**

From 2002 to 2019, a total of 12,312 patients underwent EVAR. The primary outcome was AAA-related mortality, with follow-up until December 31, 2019. Using 5 ML algorithms, we developed a model comprising 34 variables. Model performance was assessed using the time-dependent C-index and Brier score. Variable importance was evaluated through permutation-based and partial dependent plots.

**Results:**

The stacking ensemble model showed the best predictive performance among the tested models (time-dependent C-index: 0.759 at 30 days, 0.716 at 365 days). The time-dependent Brier scores generally increased slightly over time but remained stable across all ML algorithms. Important predictors included age, smoking status, duration between diagnosis and surgery, household income, renal function, and blood pressure. Variable importance differed over time, and each predictor presented a nonlinear relationship with AAA-related mortality risk.

**Conclusion:**

The stacking ensemble ML model for time-to-event prediction identified dynamic, time-varying changes in predictor importance, providing improved risk stratification and phase-specific management after EVAR.

## Introduction

Abdominal aortic aneurysm (AAA) is defined as a pathological dilation of the abdominal aorta and is associated with a high mortality rate, approaching 80% upon rupture [1–3]. Currently, no effective pharmacological treatment exists to prevent the progression or rupture of AAA; therefore, surgical intervention remains the primary therapeutic strategy [1,4,5]. Surgical intervention is generally recommended for AAAs with a diameter of ≥ 5.5 cm in males and ≥ 5.0 cm in females [5–7]. The two primary surgical options are open aneurysm repair (OAR) and endovascular aneurysm repair (EVAR) [8]. Recently, EVAR has gained widespread popularity, accounting for up to 80% of AAA repair, due to its minimally invasive nature and lower early postoperative mortality compared to OAR [6,8,9]. Despite these advantages, EVAR remains associated with important long-term complications, including endoleaks and late aneurysm rupture, which are serious enough to necessitate lifelong surveillance [7,10,11].

Mortality outcomes following EVAR are influenced by numerous factors, including demographic characteristics, anthropometric measures, and comorbidities [12–15]. In addition to well-established risk factors such as advanced age, female sex, and renal disease, socioeconomic factors have also emerged as potential contributors to AAA-related mortality. Low household income, in particular, has been consistently linked to poorer AAA-related outcomes [16]. Previous research indicates that, compared to privately insured patients, those with low income have a 47% higher mortality rate following AAA surgery, and uninsured patients exhibit a 102% higher mortality rate [15]. Moreover, patients with low BMI have also been reported to have increased mortality following EVAR [13,14]. Therefore, evaluating the impact of socioeconomic and anthropometric factors on long-term survival prediction after EVAR is clinically important.

Currently, a well-established and widely accepted model for predicting long-term prognosis after EVAR is lacking, and existing models have limited clinical utility [17]. A systematic review of 13 risk prediction models for AAA treatment outcomes highlighted numerous methodological limitations, variable predictive performance across different populations, and heterogeneity in predictor variables [18]. Notably, most existing models included heterogeneous patient populations combining EVAR and OAR recipients without adequately distinguishing between the two surgical techniques [18]. Although recent studies have attempted to predict long-term outcomes specifically in EVAR populations, these have predominantly relied on conventional statistical approaches, such as Cox proportional hazards regression [9,19], While these traditional methods offer intuitive interpretations and rapid assessments, they assume proportional hazards and linear relationships among variables, thus limiting their capacity to identify complex, non-linear interactions [20]. Moreover, these methods struggle to effectively address the dynamic, time-dependent nature of risk, in which the relative contribution of prognostic factors may change throughout different phases of disease progression [21].

Recent advances in machine learning (ML) have offered new opportunities to enhance predictive accuracy by accommodating complex, nonlinear relationships and simultaneously integrating multiple patient characteristics without restrictive statistical assumptions [22,23]. Particularly, ensemble ML techniques, like stacking [24], aggregate predictions from multiple models showing superior performance by reducing prediction errors and addressing issues of bias and variance [25,26]. Additionally, ML-based survival analyses facilitate dynamic evaluation of risk over time, providing deeper insights into how prognostic factors influence patient outcomes across different stages of disease progression [27,28]. However, most prior ML-based studies of AAA have focused primarily on classification tasks, limiting the ability to evaluate temporal variations in risk and predictor importance [17,29].

Recently, our group conducted a large-scale national cohort study of patients undergoing EVAR using the Korean NHIS database; in this study, we demonstrated that insurance type was independently associated with AAA-related mortality, even after adjusting for demographic, clinical, and laboratory variables [30]. Specifically, Medical Aid patients exhibited significantly higher AAA-related mortality compared to National Health Insurance patients, as shown by Kaplan–Meier survival analyses and multivariable Cox regression models with propensity score matching [30]. These findings underscore the prognostic value of structured real-world data, even in the absence of detailed anatomical information.

Building on this finding, the present study aimed to develop a time-to-event predictive model utilizing stacking-based ensemble ML techniques within the Korean nationwide EVAR cohort. By evaluating the time-varying importance of predictive variables, we seek to identify the dynamic contributions of key risk factors to mortality and to support risk stratification and data-driven decision-making in large-scale EVAR populations.

## Methods

### Data source and study population

This study used data from the Korean National Health Insurance System (NHIS) database, which provides universal healthcare coverage to all South Korean residents. Data access was approved by the NHIS Institutional Review Board and can be requested by qualified researchers via the Korean National Health Insurance Sharing Service (NHISS) (https://nhiss.nhis.or.kr). The NHIS dataset used in this study was accessed for research purposes on 31 March 2025. All data provided to the research team were fully anonymized before access, and no author had access to any information that could identify individual participants during or after data extraction.

**Fig 1:**
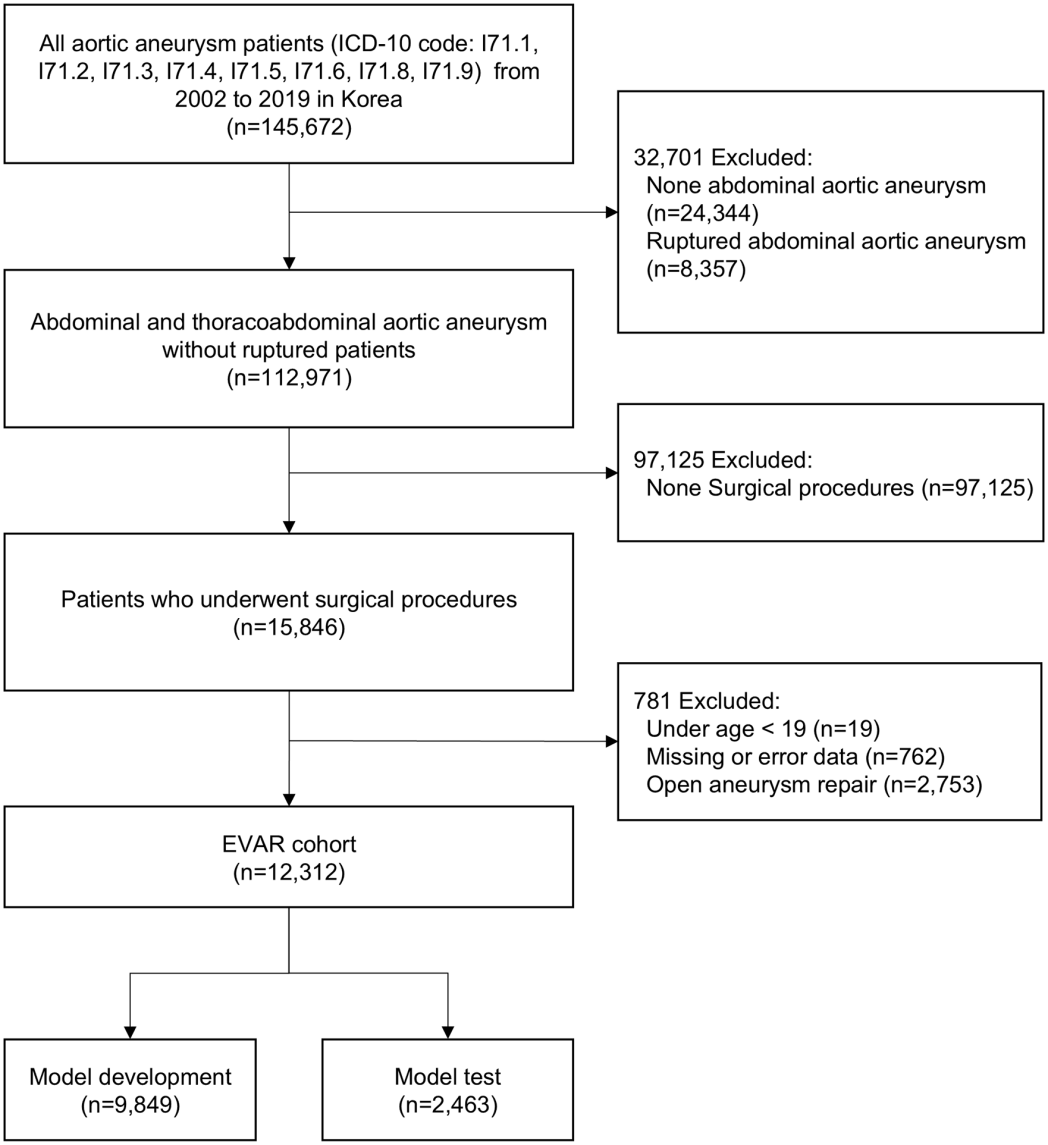
Flowchart of the study population.

We extracted data from the Korean NHIS database for all patients newly diagnosed with aortic aneurysms from 2002 to 2019 (n = 145,672). Patients diagnosed with aneurysms other than AAA (n = 24,344) and those presenting with ruptured AAA (n = 8,357) were excluded. We further restricted the cohort to patients with AAA who underwent surgical intervention, specifically EVAR. Consequently, patients who did not undergo surgical intervention (n = 97,125), those younger than 19 years (n = 19), patients with missing or erroneous data (n = 762), and those who initially underwent open aneurysm repair (n = 2,753) were sequentially excluded. The final cohort comprised 12,312 patients who initially underwent EVAR. This cohort was randomly divided into a development set (80%; n = 9,849) and a test set (20%; n = 2,463) for subsequent analysis (Fig 1).

### Data collection and definition

A total of 34 variables were extracted from the NHIS database. As predictors of survival in patients with EVAR, 13 variables of patient characteristics, 10 variables of clinical, and 11 variables of laboratory were derived. From the NHIS database, we collected the following patient characteristics: age, sex, insurance type, residential area, household income quintile, smoking status, regular exercise, height, weight, body mass index, waist circumference, and family history of hypertension or diabetes mellitus (DM). We also gathered information on clinical characteristics (i.e., systolic and diastolic blood pressure, the duration from diagnosis to surgery, and comorbidities such as hypertension, DM, dyslipidemia, coronary artery disease, chronic kidney disease, cerebrovascular disease, and malignant neoplasms and chronic) and laboratory findings (i.e., hemoglobin, fasting blood sugar [FBS], total cholesterol, aspartate transaminase [AST], alanine transaminase [ALT], triglycerides, gamma-glutamyltransferase [GGT], high-density lipoprotein [HDL], low-density lipoprotein [LDL], creatinine, and glomerular filtration rate [GFR]). The Republic of Korea’s universal health insurance system includes two main insurance services―national health insurance (NHI) and Medical Aid. Residential area was classified as metropolitan, urban, or rural. Household income quintile was determined based on health insurance contributions, which reflect income levels, by dividing the population into five equal groups (20% each). The lowest quintile (Q1) represented the lowest 20% of income earners, while the highest quintile (Q5) represented the top 20%. Medical Aid recipients were classified separately as income quintile 0. Regular exercise was classified based on whether the individual engaged in moderate exercise ≥ 5 days/week or vigorous exercise ≥ 3 days/week. Lifestyle and laboratory data were derived from national health screenings. Details on variable definitions, proportions of missing data, and imputation strategies are provided in S1 Table. Comorbidities were defined using ICD-10 codes (S2 Table).

### Outcome

The primary outcome of this study was time to AAA-related mortality, with follow-up until December 31, 2019. AAA-related mortality was defined using specific cause-of-death codes from death records, as listed in S2 Table. AAA-related mortality was primarily defined as cases where the cause of death diagnosis included the I71 code, which represents aortic aneurysm and dissection. Additionally, AAA-related mortality included cases where at least one of the following associated diagnosis codes was reported, as these represent major complications linked to AAA-related mortality: K66 (including intraperitoneal hemorrhage), R58 (hemorrhage NOS [not elsewhere classified]), R57 (shock, unspecified), and I77 (including rupture of an artery) [30,31]. Survival time was calculated from the date of the EVAR procedure.

### Data analysis

Continuous variables were expressed as the mean with standard deviation. Frequency and percentage were expressed for categorical variables. To compare two different groups, we used an independent t-test for continuous variables and a chi-squared test for categorical variables. For survival analysis, time-to-event data were constructed with AAA-related mortality coded as 0 (censored) or 1 (event). Participants who did not experience AAA-related mortality during the follow-up period were treated as right-censored observations at the time of loss to follow-up. All statistical analyses were performed using SAS Enterprise Guide 7.15 (SAS Institute Inc., Cary, NC, USA) or Python 3.8.16 (Python Software Foundation, Beaverton, OR, USA). All the tests of statistical significance were two-tailed, and statistical significance was set at p-value < 0.05.

### Model development and validation

Missing data were assumed to be missing at random and imputed using standard approaches: mode imputation for binary and categorical variables, and mean imputation for continuous variables. Continuous variables were normalized using min-max scaling, and categorical variables were transformed via one-hot encoding. To minimize the risk of overfitting, we performed 5-fold cross-validation to evaluate both the discrimination and calibration performance of our models. Specifically, the final study cohort was randomly divided into a training set (80%; n = 9,849) for model development and a hold-out test set (20%; n = 2,463) for model test. We applied a stacking ensemble approach, integrating four ML-based survival models: Cox proportional hazards, gradient boosting survival analysis, random survival forests, and extra survival trees. This stacking ensemble approach was chosen over single-model or homogeneous ensemble techniques (e.g., bagging or boosting) because it enables the combination of heterogeneous survival learners with diverse modeling assumptions [27,28]. By combining Cox proportional hazards models with tree-based survival algorithms, the stacking framework leverages their complementary strengths through a meta-learner, which can be advantageous in scenarios where proportional hazards assumptions and nonlinear effects coexist.

These base models were combined using a stacking method based on the time-dependent concordance index (time-dependent c-index) to enhance predictive performance. Hyperparameters were optimized within the development set using cross-validation. Finally, the optimized model was independently validated using the hold-out test set to confirm its generalizability. We developed both base and stacking ensemble ML models using the Scikit-survival (version 0.24.0) library on Python (version 3.8.16) [32].

### Model performance

The utility of a survival model should be assessed by how well the model discriminates between predicted risks and by how well the model is calibrated. In this study, we employed the time-dependent C-index and Brier score adjusted for the right-censored patients to assess the model’s discriminative and predictive performances, respectively. The time-dependent C-index gauges the model’s effectiveness in discriminating individual risks at different time points, particularly in the presence of censored data. The time-dependent Brier score assesses the accuracy of a survival model’s predictions regarding the distribution of the observed events at different time points of interest.

For assessing the importance of variables, we first utilized the permutation-based variable importance score, defined as the decrease in the model’s performance when the value of each variable is randomly permuted. As this random permutation breaks the dependency of the given variable on the target outcome, the resulting performance drop indicates the model’s reliance on that particular variable. For assessing performance, we employed the time-dependent C-index, highlighting that the most significant variable causes the most substantial drop in the model’s discriminative power. Also, we utilized the partial dependence plots, which show the impact of certain variables on the outcome prediction by marginalizing the remaining variables except for the chosen variable. Specifically, we determined the variable importance score for each variable by averaging the predicted value by altering the variable value from its minimum to its maximum (for continuous features), from 0 to 1 (for binary features), and from the mode to each category (for categorical features).

### Ethical statement

The study was conducted according to the ethical principles outlined in the Declaration of Helsinki. This study was approved by the institutional review board (IRB) of Boramae Medical Center (IRB No. 07-2025-4). Obtaining written informed consent was waived by the IRB due to the routine nature of the information collected and the retrospective nature of the study design. All data were anonymous and de-personalized. The study adhered to the Transparent Reporting of a Multivariable Prediction Model for Individual Prognosis or Diagnosis + Artificial Intelligence (TRIPOD+AI) checklist guidelines [33].

## Results

### Participant characteristics

The baseline characteristics of the study patients are presented in Table 1. During the median follow-up period of 2.8 years (interquartile range: 1.1–5.4 years), a total of 740 patients (6.0%) experienced AAA-related mortality. The average age of the total patient population was 72.8 years, with 10,173 (82.6%) being male. Mean age was significantly different between the event group and censored group (75.1 vs 72.7 years; p < 0.001). The event group had significantly higher proportions of females and Medical Aid. Additionally, the event group had significantly lower weight and height compared to the censored group (p < 0.001). The proportion of patients engaging in regular exercise was also lower in the event group (p < 0.001). Among clinical variables, hypertension was the most prevalent, observed in 24.3% of total patients. Comorbidities did not show a statistically significant difference between the event and censored groups. However, systolic and diastolic blood pressure were significantly higher in the event group compared to the censored group. Regarding laboratory variables, hemoglobin levels were significantly higher in the event group compared to the censored group (13.5 vs 13.9 g/dL; p < 0.001). In terms of lipid-related parameters, no significant differences were observed between the two groups, except for triglycerides. Kidney function was significantly lower in the event group than in the censored group.

**Table 1:** Baseline Characteristics of Patients who Underwent EVAR for Abdominal Aortic Aneurysm.

Characteristics		Total (n = 12,312)		Event (n = 740)	Censored (n = 11,572)	*p-value*
**Patient characteristics**						
Age (years)			72.84 (8.74)	75.11 (8.61)	72.70 (8.73)	<0.001
Sex, n (%)						
	Male		10173 (82.6)	548 (74.0)	9625 (83.2)	<0.001
	Female		2139 (17.4)	192 (26.0)	1947 (16.8)	
Insurance type, n (%)						
	NHI		11434 (92.9)	667 (90.1)	10767 (93.0)	0.004
	Medical Aid		878 (7.1)	73 (9.9)	805 (7.0)	
Residential area, n (%)						
	Metropolitan		5505 (44.8)	346 (46.9)	5159 (44.7)	0.494
	Urban		5105 (41.5)	296 (40.1)	4809 (41.6)	
	Rural		1681 (13.7)	96 (13.0)	1585 (13.7)	
Household income quintile, n (%)						0.036
	Medical Aid		878 (7.3)	73 (10.2)	805 (7.1)	
	Q1		1540 (12.7)	88 (12.3)	1452 (12.8)	
	Q2		1259 (10.4)	82 (11.4)	1177 (10.3)	
	Q3		1585 (13.1)	92 (12.8)	1493 (13.1)	
	Q4		2361 (19.5)	140 (19.5)	2221 (19.5)	
	Q5		4477 (37.0)	243 (33.8)	4234 (37.2)	
Smoking, n (%)						
	Never		3687 (30.0)	233 (31.5)	3454 (29.8)	<0.001
	Ex-smoker		3100 (25.2)	137 (18.5)	2963 (25.6)	
	Current		3182 (25.8)	154 (20.8)	3028 (26.2)	
	Unknown		2343 (19.0)	216 (29.2)	2127 (18.4)	
Regular exercise, n (%)			3461 (28.1)	150 (20.27)	3311 (28.61)	<0.001
Height (cm)			164.68 (7.95)	162.37 (8.56)	164.81 (7.90)	<0.001
Weight (kg)			65.95 (10.52)	63.77 (11.28)	66.08 (10.46)	<0.001
BMI (kg/m^2^)			24.26 (3.07)	24.11 (3.39)	24.27 (3.05)	0.306
Waist circumference (cm)			86.75 (8.55)	86.22 (9.46)	86.78 (8.50)	0.225
Family history of hypertension, n (%)			1119 (9.1)	43 (5.8)	1076 (9.3)	0.002
Family history of diabetes mellitus, n (%)			447 (3.6)	11 (1.5)	436 (3.8)	0.002
**Clinical variables**						
SBP (mmHg)			130.10 (16.47)	132.23 (17.54)	129.98 (16.40)	0.004
DBP (mmHg)			78.94 (10.85)	80.60 (11.27)	78.85 (10.82)	0.001
Duration from diagnosis to surgery (days)			505.55 (901.04)	567.53 (936.73)	501.59 (898.60)	0.063
Hypertension, n (%)			2996 (24.3)	165 (22.3)	2831 (24.5)	0.198
Diabetes mellitus, n (%)			471 (3.8)	24 (3.2)	447 (3.9)	0.451
Dyslipidemia, n (%)			970 (7.9)	47 (6.4)	923 (8.0)	0.128
Coronary artery disease, n (%)			459 (3.7)	18 (2.4)	441 (3.8)	0.069
Chronic kidney disease, n (%)			96 (0.8)	5 (0.7)	91 (0.8)	0.907
Cerebrovascular disease, n (%)			72 (0.6)	7 (1.0)	65 (0.6)	0.205
Malignant neoplasms, n (%)			334 (2.7)	15 (2.0)	319 (2.8)	0.286
**Laboratory variables**						
Hemoglobin (g/dL)			13.89 (1.63)	13.46 (1.70)	13.91 (1.62)	<0.001
FBS (mg/dL)			103.35 (23.64)	103.45 (28.66)	103.34 (23.32)	0.929
Total cholesterol (mg/dL)			187.32 (42.58)	187.64 (43.17)	187.30 (42.55)	0.859
AST (U/L)			26.36 (15.37)	25.35 (17.89)	26.41 (15.21)	0.179
ALT (U/L)			22.84 (16.03)	20.20 (13.75)	22.99 (16.13)	<0.001
Triglyceride (mg/dL)			141.48 (85.74)	131.28 (63.25)	142.02 (86.73)	0.001
GGT (U/L)			41.00 (56.26)	39.62 (65.90)	41.08 (55.68)	0.616
HDL (mg/dL)			47.24 (12.23)	47.15 (12.49)	47.24 (12.21)	0.887
LDL (mg/dL)			109.74 (37.93)	110.32 (36.95)	109.71 (37.98)	0.755
Creatinine (mg/dL)			1.17 (0.89)	1.32 (1.19)	1.16 (0.87)	0.011
GFR (mL/min/1.73)			72.02 (22.85)	62.33 (23.28)	72.44 (22.74)	<0.001

EVAR, endovascular aneurysm repair; NHI, National Health Insurance; BMI, body mass index; SBP, systolic blood pressure; DBP, diastolic blood pressure; FBS, fasting blood sugar; AST, aspartate transaminase; ALT, alanine transaminase; GGT, gamma-glutamyltransferase; HDL, high-density lipoprotein; LDL, low-density lipoprotein; GFR, glomerular filtration rate.

**Fig 2:**
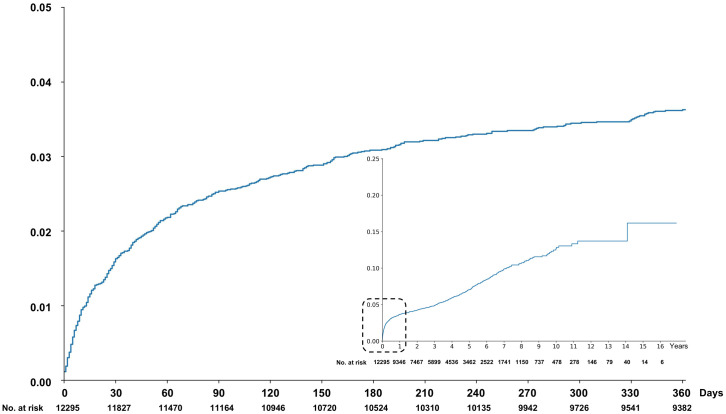
Cumulative incidence of AAA-related mortality in patients who underwent EVAR.

Fig 2 is a Kaplan-Meier curve representing the cumulative AAA-related mortality rate. The cumulative AAA-related mortality rate increased sharply within the first 60 days after EVAR. The cumulative mortality rate was 1.6% at 1 month, 3.1% at 6 months, 3.6% at 1 year, 4.2% at 2 years, and 7.0% at 3 years.

### Performance of the prediction model

**Fig 3:**
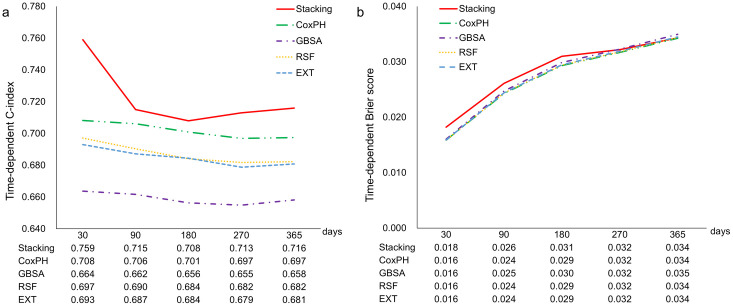
Comparison of time-dependent C-index and Brier score in prediction models. **(a)** Comparison of time-dependent C-index in prediction models. **(b)** Comparison of time-dependent Brier score in prediction models.

To evaluate the performance of various survival prediction models for patients who underwent EVAR, a comparative analysis was conducted using the time-dependent C-index and the time-dependent Brier score. Fig 3a illustrates a comparative analysis of the time-dependent C-index for multiple predictive models, including the stacking-based ensemble model (Stacking), Cox Proportional Hazards (CoxPH), Gradient Boosting Survival analysis (GBSA), Random Survival Forest (RSF), and Extra Survival Trees (EXT). This evaluation was conducted at specific time points (30, 90, 180, 270, and 365 days) during the validation of the test set.

Stacking consistently demonstrated superior time-dependent C-index values at each evaluated time point, beginning with the highest value of 0.759 at 30 days and declining to 0.716 at 365 days. Fig 3b presents a comparative analysis of the time-dependent Brier scores for the same predictive models. Across the evaluation period, an overall increasing trend in the time-dependent Brier scores was observed. All predictive models showed stable and consistent time-dependent Brier score values across the measured time points, with relatively minimal variation.

### Variable importance

**Fig 4:**
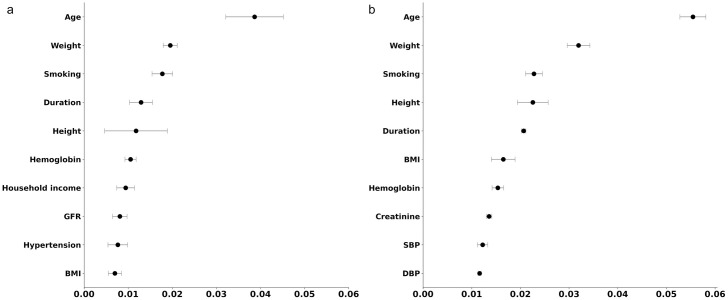
Variable importance in a stacking ensemble model for AAA-related mortality. **(a)** Top-ranked predictor importance for 30-day mortality in the stacking ensemble model using permutation feature importance. **(b)** Top-ranked predictor importance for 1-year mortality in the stacking ensemble model using permutation feature importance.

The top-ranked variable importance over time in Stacking, the model with the highest time-dependent C-index, was assessed using the permutation-based variable importance (S3 Table). At 30 days, the variable with the greatest importance was age, followed by weight, smoking, duration from diagnosis to surgery, and height (Fig 4a). At 365 days, age, weight, and smoking still remained the most important variables, while the importance of BMI, creatinine, systolic blood pressure (SBP), and diastolic blood pressure (DBP) increased (Fig 4b). In contrast, household income, GFR, and hypertension were no longer among the top 10 most important variables.

**Fig 5:**
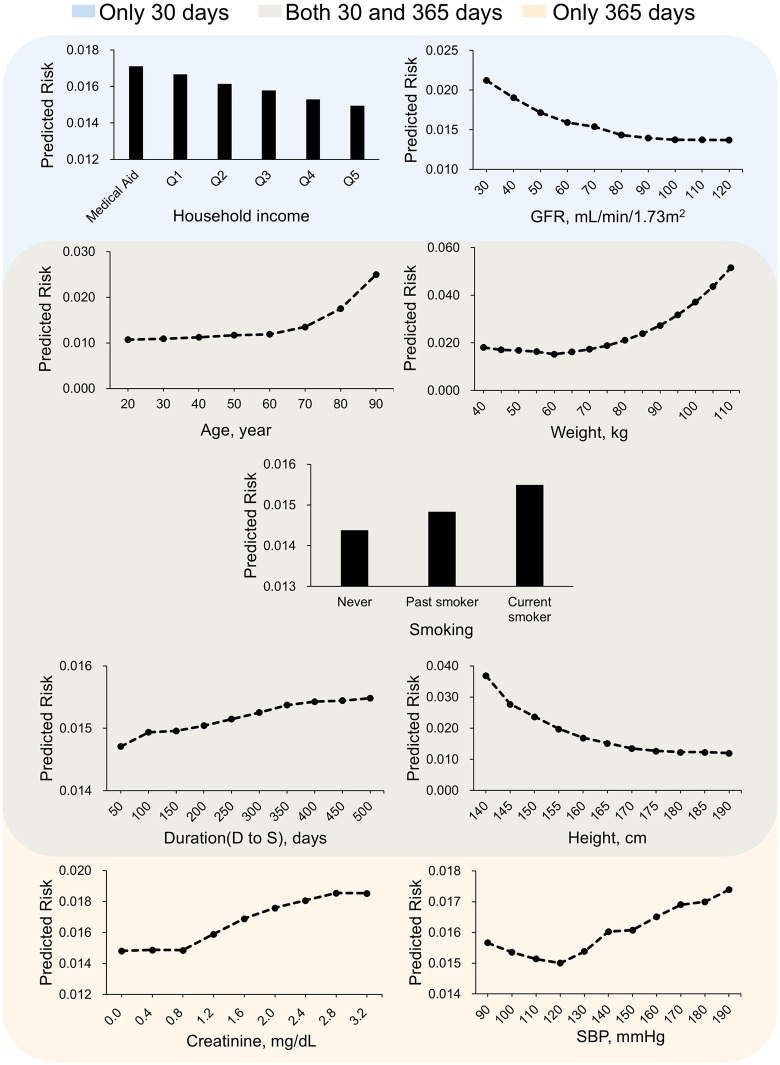
Partial dependence plots showing key predictors of AAA-related mortality in patients who underwent EVAR. Predictors are color-coded by their importance for 30-day mortality (blue), 1-year mortality (orange), or both (gray).

We analyzed the influence of top-ranked variables on AAA-related mortality at 30 and 365 days. Fig 5 illustrates the relationship between these top-ranked variables and AAA-related mortality using partial dependence plots. To enhance interpretability, variables were grouped into three categories based on their temporal importance: variables important only at 30 days are shown in blue-shaded panels, those important only at 365 days in orange-shaded panels, and those important at both time points in gray-shaded panels.

Specifically, age showed a sharp increase in AAA-related mortality risk in patients aged 70 years or older. Weight showed the lowest risk around 60 kg, with the risk increasing both above and slightly below this value. Smoking status showed that current smokers had the highest risk of AAA-related mortality. A longer duration from diagnosis to surgery was associated with a higher risk. Height showed an inverse relationship with mortality risk, with shorter individuals having a higher risk. Lower household income was linked to higher risk, with the highest risk observed in the Medical Aid group. GFR values below 80 mL/min/1.73 m^2^ were associated with increased risk. AAA-related mortality risk increased progressively with creatinine levels above 0.8 mg/dL. SBP showed the lowest predicted risk around 120 mmHg. Risk increased at both lower and higher SBP levels, with a more notable increase at higher SBP.

## Discussion

In the present study, we developed a stacking ensemble ML model to predict AAA-related mortality among patients undergoing EVAR, applying a time-to-event analysis framework. A key strength of this approach was the ability to evaluate how the relative importance of variables changed over different time points after EVAR, highlighting specific predictors of short-term (30-day) and long-term (365-day) AAA-related mortality. In addition, several predictors—such as age, smoking status, and the interval between diagnosis and surgery—showed consistent influence across both periods, representing persistent risk factors that were associated with outcomes regardless of time. Furthermore, our study employed partial dependence plots, which illustrated important nonlinear relationships between variables and mortality risk—relationships not typically captured by traditional statistical modeling methods. Our methodological framework provided valuable insights into both the temporal dynamics and nonlinear nature of baseline predictors, thereby supporting phase-specific and continuous risk stratification as well as data-driven clinical decision-making in EVAR-treated AAA patients. These findings extend prior evidence that socioeconomic and clinical variables derived from insurance claims data can yield clinically relevant prognostic information, even in the absence of detailed anatomical characteristics [30], highlighting the potential of real-world data to inform clinically meaningful temporal dynamics of risk evaluation.

Early postoperative mortality appeared to be more strongly associated with household income and baseline renal function as estimated by GFR. Lower household income was associated with higher 30-day AAA-related mortality, which may reflect differences in healthcare utilization, health behaviors, and broader socioeconomic circumstances [30]. For example, lower household income could be associated with variability in healthcare utilization levels [34,35], less favorable health behaviors such as smoking or inadequate weight management [36], and broader systemic barriers including limited health literacy, reduced access to continuous care, and difficulties engaging with healthcare services [37]. In the context of EVAR, these socioeconomic disparities may be associated with differences in access to care, medication adherence, and availability of recovery resources during the early postoperative period. In addition, lower estimated GFR was associated with early postoperative mortality, consistent with reduced physiological reserve. Unlike serum creatinine, which is affected by patient characteristics such as muscle mass, sex, and age, GFR provides a more direct estimate of renal filtration capacity and tends to decline earlier in the course of renal dysfunction [38]. This characteristic may account for its stronger association with early postoperative mortality observed in this study. Collectively, these findings suggest that early mortality after EVAR was more strongly associated with patient vulnerability and socioeconomic disadvantage, supporting the potential value of early identification and perioperative optimization in socially or medically high-risk groups.

At 365 days after EVAR, baseline serum creatinine and SBP were identified as two of the strongest predictors associated with long-term mortality. Creatinine showed a nonlinear association with mortality risk, remaining relatively stable within the normal range but gradually increasing at higher levels. This pattern may not directly indicate ongoing renal injury but rather reflect pre-existing mild kidney dysfunction or general health vulnerability present before EVAR [38,39]. Because serum creatinine is influenced by factors such as muscle mass, age, and overall metabolic status [38], elevated levels may serve as a proxy marker of broader systemic vulnerability rather than a direct causal factor of late mortality. Nonetheless, this finding aligns with prior studies indicating that even mild renal impairment before surgery is associated with poorer long-term outcomes after EVAR [38]. In contrast, GFR typically declines earlier in renal dysfunction, while creatinine tends to rise later [38], suggesting that these measures provide complementary but distinct clinical information about renal and overall health status at baseline.

SBP also exhibited a nonlinear relationship with mortality risk, with the lowest risk observed around 120 mmHg and increasing risk at both higher and lower SBP values. This U-shaped association suggests that sustained hypertension may contribute to vascular and graft-related stress, whereas excessive lowering of SBP may be associated with reduced organ perfusion and impaired recovery capacity. Prior evidence indicating that tight SBP control (<130 mmHg) is associated with a reduced incidence of type II endoleaks after EVAR further supports the clinical relevance of balanced hemodynamic management [40]. Collectively, these findings suggest that long-term mortality after EVAR is associated with both chronic renal vulnerability and nonlinear hemodynamic patterns, emphasizing the importance of continuous monitoring and optimization beyond the perioperative period.

Across both early and late postoperative phases, age, smoking, and the interval between diagnosis and surgery were consistently associated with AAA-related mortality. Age was identified as a key predictor, with a sharply increased risk observed beyond approximately 70 years. This pattern aligns with prior prognostic models for EVAR-treated patients, which stratified age into < 70, 70–74.9, 75–79.9, and ≥ 80 years, assigning progressively greater weights with increasing age [19]. Although age alone may not fully capture physiological heterogeneity, the consistently higher risk among older patients is consistent with a cumulative burden related to vascular aging, frailty, and comorbid burden that extends throughout the postoperative course. Smoking was also consistently associated with higher mortality, reinforcing its persistent role as a major modifiable risk factor contributing to both early and late adverse outcomes after EVAR [41].

A longer interval between AAA diagnosis and EVAR was also associated with higher AAA-related mortality. In this cohort, the mean duration from diagnosis to surgery was approximately 17 months. Although aneurysm size at diagnosis was not available, this finding may indicate that delayed surgical intervention reflects several overlapping factors rather than a single mechanism. First, some patients may have experienced rapid aneurysmal progression after diagnosis [42], particularly if their initial aneurysm was of moderate size. Second, delays in surgical referral or coordination within the healthcare system may have been associated with postponed intervention. Third, certain patients may have deferred surgery until symptom onset, leading to urgent or high-risk procedures once aneurysm expansion became clinically significant [43]. Taken together, these interpretations suggest that a prolonged diagnosis-to-surgery interval may represent a composite marker of inadequate follow-up, delayed clinical decision-making, or symptom-driven emergency repair, and was associated with worse postoperative outcomes.

A principal finding of this study is the time-varying nature of predictor importance for mortality after EVAR. The predictors of early (30-day) and late (365-day) mortality differed substantially, indicating potentially distinct clinical mechanisms [7,44]. Early mortality was more strongly associated with perioperative vulnerability—such as household income and reduced GFR— whereas late mortality showed stronger associations with chronic factors, including creatinine levels and BP control. These findings highlight that patient management after EVAR may benefit from a phase-specific approach, combining targeted perioperative care with long-term surveillance and chronic disease management.

This study applied a time-to-event stacking ensemble framework that enabled the identification of phase-specific and time-varying risk patterns for AAA-related mortality. By combining multiple base learners within a survival modeling structure, the approach allowed evaluation of how each predictor’s contribution changed over time, without relying on restrictive assumptions such as proportional hazards or linearity [27,28]. This framework also captured nonlinear relationships and interactions among predictors, providing a more flexible representation of the complex, multidimensional processes underlying post-EVAR outcomes. Similarly, previous research indicated that stacking ensemble techniques leverage the predictions of constituent models as input for a meta-learner, which can enhance predictive performance compared to other ensemble techniques [27,45]. However, as the model was not externally validated, the generalizability of these phase-specific patterns should be interpreted with caution. To enhance interpretability, permutation feature importance and partial dependence plots were used to visualize how each predictor was associated with model output [27,46]. Permutation analysis identified the variables that had the impact on prediction performance at 30 and 365 days, while partial dependence plots illustrated the nonlinear and time-dependent effects of key predictors such as household income, creatinine, and SBP. Together, these methods supported a data-driven understanding of how the relative influence of clinical and socioeconomic factors evolves across follow-up [27,46,47], representing an incremental extension over conventional survival analyses.

There are several limitations to this study. First, as a retrospective study based on nationwide Korean population data, it is subject to various biases, including selection bias. We therefore included all available data to minimize these biases. Second, identifying AAA and other risk factors solely based on diagnostic codes may have introduced inaccuracies. There is potential for outcome misclassification because AAA-related mortality was defined using cause-of-death codes without clinical adjudication, which may bias the estimated event rates. Nevertheless, most deaths were coded as I71, consistent with the definitions of AAA-related mortality commonly used in prior studies. Third, this study is subject to the inherent limitations of administrative data. Detailed anatomical and physiological information relevant to EVAR outcomes—such as aneurysm diameter, neck morphology, calcification, endoleak status, sarcopenia, cardiopulmonary fitness, or cardiac function—was unavailable, as were psychosocial factors like stress. Accordingly, our findings should be interpreted as risk predictions based on routinely collected administrative variables, rather than as substitutes for anatomical or procedure-specific risk assessments. Fourth, missing values in several clinical and laboratory predictors were addressed using simple mean imputation. For variables with substantial missingness, this method may reduce variability and affect the relative importance ranking of predictors. In our analyses, renal function markers (e.g., GFR and creatinine) were consistently identified as influential predictors despite potential variance attenuation. Future studies should consider alternative missing-data techniques such as multiple imputation to further assess the stability of predictor importance and model performance. Fifth, this study relied on internal validation using a hold-out dataset and did not include external validation with independent cohorts. Therefore, the reported discrimination and calibration may not fully represent the model’s performance across different clinical settings, time periods, or coding practices. Consequently, the model’s transportability requires further confirmation. Lastly, because the study population consisted entirely of Koreans, the model’s performance and the relative influence of predictors may vary in populations with different baseline risk profiles and healthcare systems, highlighting the need for external validation in diverse, international cohorts.

## Conclusion

This study suggests the potential utility of a stacking ensemble machine learning approach for time-to-event prediction to enhance survival estimation after EVAR by identifying time-varying effects of key mortality predictors. Overall, age, smoking, and the duration between diagnosis and surgery consistently emerged as important risk factors. Household income and GFR were more strongly associated with early postoperative mortality, whereas baseline blood pressure and serum creatinine levels were key determinants of long-term outcomes. These findings indicate that ML-based approaches may support improved risk stratification and support phase-specific follow-up and management strategies for patients undergoing EVAR.

## Supporting information

S1 Table Variable information. (DOCX)

S2 Table Definitions of variables. (DOCX)

S3 Table Top-ranked variable importance over time in the prediction. (DOCX)
