# Supplementary material for: Time-to-event ensemble machine learning approach for predicting long-term survival of abdominal aortic aneurysm patients undergoing endovascular aneurysm repair
Source: PLoS One. 2026 Jun 12;21(6):e0349122. doi: 10.1371/journal.pone.0349122 (PMC13262846; doi:10.1371/journal.pone.0349122)
Supplement: S1 Table — (DOCX) [file pone.0349122.s001.docx]

**S1 Table.** Variable Information

| **Variable** | **Description** | **Type of variables** | **Value** | **Ratio of missing value** | **Imputation method** |
| --- | --- | --- | --- | --- | --- |
| **Patient characteristic** |  |  |  |  |  |
| Age, years | Age of the patients | Numeric | Value | 0.0% | N/A |
| Sex | Sex of the patients | Binary | 0 = Female  1 = Male | 0.0% | N/A |
| Insurance type | The Republic of Korea’s universal health insurance system includes two main insurance services―national health insurance and medical aid | Binary | 0 = NHI  1 = Medical aid | 0.0% | N/A |
| Residential area | Residential area was categorized based on the patient's place of residence and classified into metropolitan, urban, and rural areas according to administrative districts. | Category | 1 = Metropolitan  2 = Urban  3 = Rural | 0.1% | Mode imputation |
| Household income quintile | Household income quintile was determined based on health insurance contributions, which reflect income levels, by dividing the population into five equal groups (20% each). The lowest quintile (Q1) represented the lowest 20% of income earners, while the highest quintile (Q5) represented the top 20%. Medical Aid recipients were classified separately as income quintile 0. | Category | 0 = Medical Aid  1 = Q1  2 = Q2  3 = Q3  4 = Q4  5 = Q5 | 0.2% | Mode imputation |
| Smoking | Smoking status (never, ex-smoker, current smoker, unknown | Category | 0 = Never  1 = Past smoker  2 = Current smoker  3 = Unknown | 0.0% | N/A |
| Regular exercise | Physical activity was classified based on whether the individual engaged in moderate exercise ≥5 days/week or vigorous exercise ≥3 days/week | Binary | 0 = No  1 = Yes | 0.0% | N/A |
| Height, cm | Height of the patients | Numeric | Value | 18.1% | Mean imputation |
| Weight, kg | Weight of the patients | Numeric | Value | 18.1% | Mean imputation |
| BMI, kg/m^2^ | Body mass index of the patients | Numeric | Value | 18.1% | Mean imputation |
| Waist circumference, cm | Waist circumference of the patients | Numeric | Value | 27.4% | Mean imputation |
| Family history of hypertension | Family history of hypertension was defined based on national health screenings | Binary | 0 = No  1 = Yes | 0.0% | N/A |
| Family history of diabetes mellitus | Family history of diabetes mellitus was defined based on national health screenings | Binary | 0 = No  1 = Yes | 0.0% | N/A |
| **Clinical variable** |  |  |  |  |  |
| Hypertension | Hypertension was defined as the presence of ICD-10 codes I10, I11, I12, I13, or I15 | Binary | 0 = No  1 = Yes | 0.0% | N/A |
| Diabetes mellitus | Diabetes mellitus was defined as the presence of ICD-10 codes, E11, E12, E13, E14 | Binary | 0 = No  1 = Yes | 0.0% | N/A |
| Dyslipidemia | Dyslipidemia was defined as the presence of ICD-10 code, E78 | Binary | 0 = No  1 = Yes | 0.0% | N/A |
| Coronary artery disease | Coronary artery disease was defined as the presence of ICD-10 codes, I20, I21, I22, I23, I24, I25 | Binary | 0 = No  1 = Yes | 0.0% | N/A |
| Chronic kidney disease | Chronic kidney disease was defined as the presence of ICD-10 code, N18 | Binary | 0 = No  1 = Yes | 0.0% | N/A |
| Cerebrovascular disease | Cerebrovascular disease was defined as the presence of ICD-10 codes, I60, I61, I62, I63, I64 | Binary | 0 = No  1 = Yes | 0.0% | N/A |
| Malignant neoplasms | Malignant neoplasms were defined as the presence of ICD-1 codes, C00-97 | Binary | 0 = No  1 = Yes | 0.0% | N/A |
| SBP, mmHg | Systolic blood pressure was defined based on national health screenings | Numeric | Value | 18.3% | Mean imputation |
| DBP, mmHg | Diastolic blood pressure was defined based on national health screenings | Numeric | Value | 18.3% | Mean imputation |
| Duration from diagnosis to surgery, days | Duration from diagnosis to surgery represents the time interval, measured in days, between the initial diagnosis of abdominal aortic aneurysm and the surgical procedure | Numeric | Value | 0.0% | N/A |
| **Laboratory variable** |  |  |  |  |  |
| Hemoglobin, g/dL | Hemoglobin levels were obtained from recorded values in national health screenings | Numeric | Value | 18.4% | Mean imputation |
| FBS, mg/dL | Fasting blood sugar was obtained from recorded values in national health screenings | Numeric | Value | 18.4% | Mean imputation |
| Total cholesterol, mg/dL | Total cholesterol was obtained from recorded values in national health screenings | Numeric | Value | 22.9% | Mean imputation |
| AST, U/L | Aspartate transaminase was obtained from recorded values in national health screenings | Numeric | Value | 18.4% | Mean imputation |
| ALT, U/L | Alanine transaminase was obtained from recorded values in national health screenings | Numeric | Value | 18.4% | Mean imputation |
| Triglyceride, mg/dL | Triglycerides were obtained from recorded values in national health screenings | Numeric | Value | 35.9% | Mean imputation |
| GGT, U/L | Gamma-glutamyltransferase was obtained from recorded values in national health screenings | Numeric | Value | 18.4% | Mean imputation |
| HDL, mg/dL | High-density lipoprotein was obtained from recorded values in national health screenings | Numeric | Value | 35.9% | Mean imputation |
| LDL, mg/dL | Low-density lipoprotein was obtained from recorded values in national health screenings | Numeric | Value | 39.6% | Mean imputation |
| Creatinine, mg/dL | Creatinine was obtained from recorded values in national health screenings | Numeric | Value | 31.4% | Mean imputation |
| GFR, mL/min/1.73 | Glomerular filtration rates were obtained from recorded values in national health screenings | Numeric | Value | 43.8% | Mean imputation |

N/A, not applicabl
