# Supplementary material for: Time-to-event ensemble machine learning approach for predicting long-term survival of abdominal aortic aneurysm patients undergoing endovascular aneurysm repair
Source: PLoS One. 2026 Jun 12;21(6):e0349122. doi: 10.1371/journal.pone.0349122 (PMC13262846; doi:10.1371/journal.pone.0349122)
Supplement: S2 Table — (DOCX) [file pone.0349122.s002.docx]

**S2 Table**. Definitions of Variables.

| **Variables** | | **Definition** |
| --- | --- | --- |
| AAA | ICD-10 codes, I71.4, I71.6, I71.9 | |
| EVAR | Codes M6611, M6612 | |
| Hypertension | ICD-10 codes, I10, I11, I12, I13, I15, I15 | |
| Diabetes mellitus | ICD-10 codes, E11, E12, E13, E14 | |
| Dyslipidemia | ICD-10 code, E78 | |
| CAD | ICD-10 codes, I20, I21, I22, I23, I24, I25 | |
| CKD | ICD-10 code, N18 | |
| Cerebrovascular disease | ICD-10 codes, I60, I61, I62, I63, I64 | |
| Malignant neoplasms | ICD-1 codes, C00-97 | |
| AAA-related death | ICD-10 codes, I71, I77, K66, R57, R58 | |

AAA, abdominal aortic aneurysm; OAR, open aneurysm repair; EVAR, endovascular aneurysm repair; CAD, coronary artery disease; CKD, chronic kidney disease; CT, computed tomography.
