# Supplementary material for: Time-to-event ensemble machine learning approach for predicting long-term survival of abdominal aortic aneurysm patients undergoing endovascular aneurysm repair
Source: PLoS One. 2026 Jun 12;21(6):e0349122. doi: 10.1371/journal.pone.0349122 (PMC13262846; doi:10.1371/journal.pone.0349122)
Supplement: S3 Table — (DOCX) [file pone.0349122.s003.docx]

**S3 Table**. Top-ranked Variable Importance Over Time in the Prediction Model.

| **Rank** | **30 days** | **90 days** | **180 days** | **270 days** | **365 days** |
| --- | --- | --- | --- | --- | --- |
| 1 | Age | Age | Age | Age | Age |
| 2 | Weight | Weight | Weight | Weight | Weight |
| 3 | Smoking | Smoking | Height | Smoking | Smoking |
| 4 | Duration* | Height | Smoking | Height | Height |
| 5 | Height | Duration* | Duration* | Duration* | Duration* |
| 6 | Hemoglobin | BMI | BMI | BMI | BMI |
| 7 | Income^†^ | Hemoglobin | Income^†^ | Hemoglobin | Hemoglobin |
| 8 | GFR | GFR | Hemoglobin | Income^†^ | Creatinine |
| 9 | Hypertension | Income^†^ | Creatinine | Creatinine | SBP |
| 10 | BMI | Sex | DBP | SBP | DBP |

GFR, glomerular filtration rate; ALT, alanine transaminase.

*Duration from diagnosis to surgery (in days).

^†^Householdl income quintile.
